# Cytotoxicity, Fractionation and Dereplication of Extracts of the Dinoflagellate *Vulcanodinium rugosum*, a Producer of Pinnatoxin G

**DOI:** 10.3390/md11093350

**Published:** 2013-09-02

**Authors:** Marie Geiger, Gwenaëlle Desanglois, Kevin Hogeveen, Valérie Fessard, Thomas Leprêtre, Florence Mondeguer, Yann Guitton, Fabienne Hervé, Véronique Séchet, Olivier Grovel, Yves-François Pouchus, Philipp Hess

**Affiliations:** 1 Ifremer, Laboratoire Phycotoxines, Centre Atlantique, 44311 Nantes Cedex, France; E-Mails: marie.geiger@hotmail.fr (M.G.); thomaslepretre@hotmail.com (T.L.); florence.mondeguer@ifremer.fr (F.M.); yann.guitton@univ-nantes.fr (Y.G.); fabienne.herve@ifremer.fr (F.H.); veronique.sechet@ifremer.fr (V.S.); 2 MMS EA2160, Faculté de Pharmacie, LUNAM, Université de Nantes, 44035 Nantes, France; E-Mails: olivier.grovel@univ-nantes.fr (O.G.); yves-francois.pouchus@univ-nantes.fr (Y.-F.P.); 3 Unité de Toxicologie des Contaminants, ANSES, 35302 Fougères, France; E-Mails: gwenaelle.desanglois@anses.fr (G.D.); kevin.hogeveen@anses.fr (K.H.); valerie.fessard@anses.fr (V.F.)

**Keywords:** dereplication, cyclic imine, HRMS, bioactivity, pinnatoxins

## Abstract

Pinnatoxin G (PnTX-G) is a marine toxin belonging to the class of cyclic imines and produced by the dinoflagellate *Vulcanodinium rugosum*. In spite of its strong toxicity to mice, leading to the classification of pinnatoxins into the class of “fast-acting toxins”, its hazard for human health has never been demonstrated. In this study, crude extracts of *V. rugosum* exhibited significant cytotoxicity against Neuro2A and KB cells. IC_50_ values of 0.38 µg mL^−1^ and 0.19 µg mL^−1^ were estimated on Neuro2A cells after only 24 h of incubation and on KB cells after 72 h of incubation, respectively. In the case of Caco-2 cells 48 h after exposure, the crude extract of *V. rugosum* induced cell cycle arrest accompanied by a dramatic increase in double strand DNA breaks, although only 40% cytotoxicity was observed at the highest concentration tested (5 µg mL^−1^). However, PnTX-G was not a potent cytotoxic compound as no reduction of the cell viability was observed on the different cell lines. Moreover, no effects on the cell cycle or DNA damage were observed following treatment of undifferentiated Caco-2 cells with PnTX-G. The crude extract of *V. rugosum* was thus partially purified using liquid-liquid partitioning and SPE clean-up. *In vitro* assays revealed strong activity of some fractions containing no PnTX-G. The crude extract and the most potent fraction were evaluated using full scan and tandem high resolution mass spectrometry. The dereplication revealed the presence of a major compound that could be putatively annotated as nakijiquinone A, *N*-carboxy-methyl-smenospongine or stachybotrin A, using the MarinLit™ database. Further investigations will be necessary to confirm the identity of the compounds responsible for the cytotoxicity and genotoxicity of the extracts of *V. rugosum*.

## 1. Introduction

**Figure 1:**
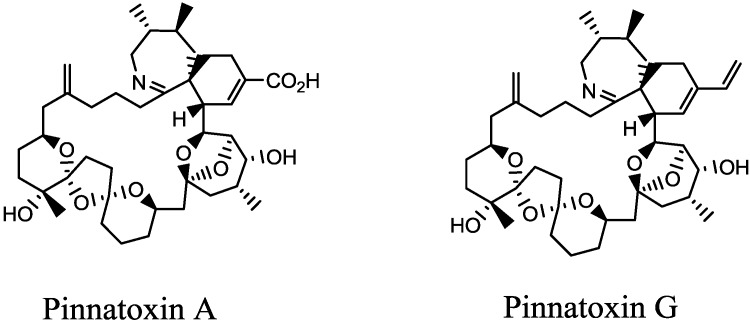
Structures of Pinnatoxin A (PnTX-A) and -G (PnTX-G). PnTX-A is a shellfish metabolite of the algal PnTX-G, with the double bond in the side chain oxidised to form a carboxylic acid group. PnTXs also contain a cyclic imine functional group.

Shellfish belonging to the genus *Pinna* had first been implicated in food poisoning in China in 1990 [1]. Pinnatoxin A (PnTX-A) was the first analogue described [2] (Figure 1), followed by PnTX-B, -C and -D, all isolated from viscera of *Pinna muricata* originated from Okinawa, Japan [3,4]. PnTX-E, -F and -G were discovered in oysters coming from Australia and New-Zealand, and PnTX-F and -G, which are algal metabolites, are suspected to be the metabolic precursor of all known PnTXs [5]. In Europe, PnTXs were first identified in Norway in 2011 in mussels and seawater samples [6].

In the regions affected by these contaminations, different dinoflagellate producers of PnTXs have been isolated: one organism was identified as a producer of PnTX-E and -F in New-Zealand [7], one as a producer of PnTX-G in Japan [8] and another one producing PnTX-E, -F and -G in Australia [9]. In 2011, a new species belonging to a new benthic dinoflagellate genus was isolated from a French Mediterranean lagoon (Ingril), and has been named *Vulcanodinium rugosum* [10]. This isolate has been shown to produce PnTX-G [11]. By morphological and phylogenetic comparison with the isolates from Japan, New-Zealand and Australia, *V. rugosum* has been identified as a single species producing PnTX-E, -F and -G in these regions [12]. More recently, PnTX-A and -G have been identified in mussels in Canada, with the first description of PnTX-G fatty acids esters [13].

To date, no acute human intoxication by consumption of shellfish contaminated by PnTXs has been reported, explaining the current absence of a regulation of PnTXs in Europe. However, a recent risk evaluation on cyclic imines suggested that further information is required on toxicology and distribution of these compounds [14]. Indeed, PnTXs and more generally, cyclic imines, have been demonstrated to be fast acting toxins on mammalian models. These toxins exhibit acute toxicity, with neurological symptoms appearing in an “all or nothing” manner [15]. Following intra-peritoneal (*i.p.*) injection into mice, PnTX-E exhibited an LD_50_ of between 16 and 57 µg kg^−1^, PnTX-F an LD_50_ of between 12.7 and 45 µg kg^−1^, and PnTX-G an LD_50_ of between 48 and 50 µg kg^−1^ [5,16]. At a lethal dose, these three analogues induced hyperactivity in mice during the first 10 min following the *i.p.* injection, then a strong decrease of the respiratory rate, followed by death of the animals between 30 and 50 min [5,16]. In comparison to other cyclic imines, PnTX-F and -G exhibited high oral toxicity in mice, with LD_50_s of 25 and 150 µg kg^−1^, respectively. This observation is likely to be correlated with the high hydrolytic stability of pinnatoxins at different pH in aqueous media [17], and suggested a high absorption of these toxins from the gastro-intestinal (GI) tract in mice [16]. 

Concerning *in vitro* activities, only PnTX-D has been reported to exhibit cytotoxicity on the P388 leukemia cell line [18]. Initially, it has been suggested that the mechanism of action of PnTXs was an activation of calcium channels [1]. However, this hypothesis has never been confirmed to our knowledge. PnTX-A, -E, -F and -G were then reported to block the nicotinic receptors of acetylcholine, with a major role of the spiro-imine subunit in this activity [19,20].

In this context, in order to investigate the various mechanisms of action and the extent of toxicity of PnTXs, the present work aimed at studying the *in vitro* toxicity of PnTX-G and *V. rugosum* extracts using cytotoxicity assays, investigating the DNA damage and effects on the cell cycle and trying to pinpoint bioactive compounds other than PnTX-G.

## 2. Results

### 2.1. Toxin Content of *V. rugosum* and Purification of PnTX-G

Axenic cultures of *V. rugosum* (276 mg pellet) yielded 22 mg of dried crude extract containing 10.5 µg of PnTX-G. The content of PnTX-G in this axenic biomass was somewhat less but of a similar height of order as the average over ten batches of non-axenic cultures in our laboratory (data not shown). The biomass of *V. rugosum* used for the fractionation and the toxicological evaluation had not been grown under axenic conditions as it is extremely difficult to maintain pilot-scale cultures in such conditions.

The fractionation scheme, which followed the isolation scheme as published by Selwood *et al.*, 2010 [5], allowed for separation of PnTX-G from other matrix components as PnTX-G was 40 times purer in fraction 3 of the silica fractionation step than in the crude extract, even when accounting for loss of toxin (Table 1). PnTX-G quantitatively partitioned into the DCM and the aqueous methanol (aqueous MeOH) phases in the first and second partitioning steps, respectively; the main cytotoxic components also were found in these two phases and not in the aqueous and hexane phases (data not shown). Also, fraction 2 only contained a negligible trace of PnTX-G compared to fraction 3. 

**Table 1:** Fractionation of crude extract used for toxicological assessment of *V. rugosum*. Approximately 99% and 96% of PnTX were recovered in DCM and aqueous MeOH phases during the first and second partitioning, respectively. More than 99% of PnTX-G was recovered in fraction 3 of the silica solid phase extraction (SPE) step.

	Fraction	Sample mass	PnTX-G
		(mg)	(µg)
**Crude extract**	n/a	2050	844
**Partitioning 1**	DCM phase	442	620
	Aqueous phase	1618	0.80
	Interface	168	5.80
**Partitioning 2**	Aq. MeOH phase	168	571
	Hexane phase	274	21.2
**Fractions**	F1	7.00	0.00
	F2	73.1	0.05
	F3	38.8	649
	F4	6.10	0.27
	F5	21.7	0.16

### 2.2. *In Vitro* Toxic Effects of Crude and Partitioned Extracts of *Vulcanodinium rugosum*

The incubation times of *V. rugosum* crude extract varied according to the cell line. Undifferentiated Caco-2 cells were incubated for 24 or 48 h. For Neuro2A and differentiated Caco-2 cells, incubation was 24 and 48 h, respectively. KB cells were incubated for 24, 48 or 72 h. 

**Figure 2:**
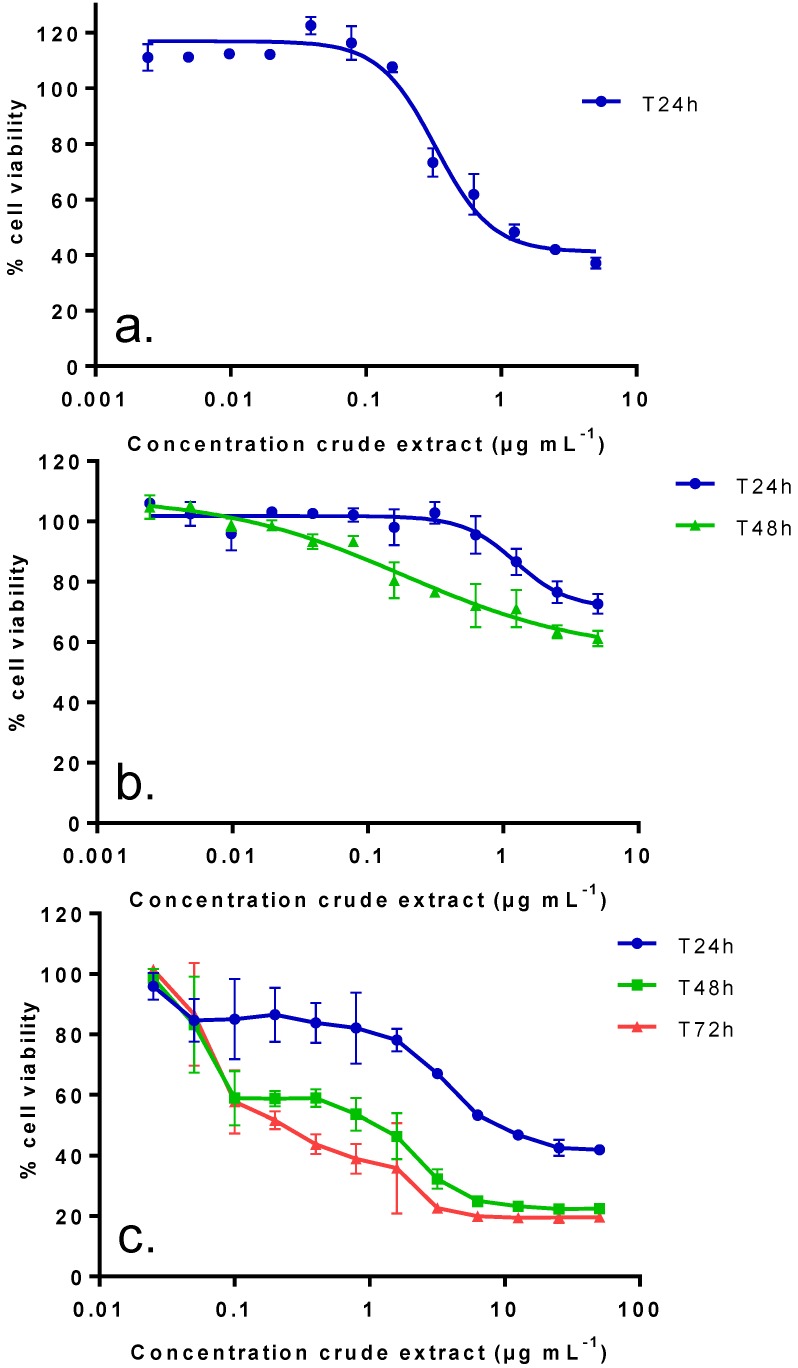
Effect of a crude extract of *V. rugosum* on Neuro2a cells (**a**), undifferentiated Caco-2 cells (**b**), and KB cells (**c**). Different exposure times were evaluated for the crude extract: 24 h for Neuro2A, 24 and 48 h for undifferentiated Caco-2 and 24, 48 and 72 h for KB cells. Data presented (mean ± SEM) were obtained from two independent experiments (duplicate wells per experiment for Neuro2A and Caco-2, triplicate wells per experiment for KB cells).

The crude extract of *V. rugosum* induced a concentration-dependent reduction of the cell viability of Neuro2A cells after only 24 h with an IC_50_ of 0.38 (0.24–0.51) µg mL^−1^ (Figure 2a). The same extract only slightly affected the viability of undifferentiated Caco2 cells, independent of exposure duration (24 or 48 h). For both exposure durations, no IC_50_ could be determined, even though a 40% cytotoxicity was observed at the highest concentration tested (5 µg mL^−1^), (Figure 2b). Moreover, no cytotoxicity was observed on differentiated Caco-2 cells after 48 h of incubation (data not shown). The crude extract of *V. rugosum* induced a time- and concentration-dependent reduction of the cell viability of KB cells (Figure 2c). The LD_50_s (with confidence interval at 95%) of *V. rugosum* crude extract on KB cells were 9 (7.8–10.2), 1 (1.09–0.91) and 0.19 (0.26–0.12) µg mL^−1^ after 24, 48 and 72 h of incubation, respectively. The extract was more potent on KB cells than on undifferentiated Caco-2 cells, and even more potent on Neuro2A cells. 

The cytotoxic activities were monitored along the partitioning steps of the crude extract of *V. rugosum*. Cytotoxicity was higher in both the DCM and the aqueous MeOH fractions than in the crude extract, both when using Neuro2A and KB cells (Table 2). However, as with the crude extract, no IC_50_ could be calculated for those two fractions on undifferentiated (Table 2) and differentiated (data not shown) Caco-2 cells, as only 40%–45% cytotoxicity was reached in undifferentiated Caco-2 cells for the highest concentrations tested (5 µg mL^−1^). 

**Table 2:** Cytotoxicity IC_50_ values (µg mL^−1^) for the crude extract, DCM and aq.MeOH fractions of *V. rugosum* crude extract (*N* = 2).

IC_50_ (µg mL^−1^): median (values obtained with two independent experiments)			
	Neuro2A cells (24 h of incubation)	KB cells (72 h of incubation)	Undifferentiated Caco-2 cells (48 h of incubation)
Crude extract	0.38 (0.24–0.51)	0.19 (0.26–0.12)	N.R. (40%)
DCM fraction	0.15 (0.14–0.15)	0.007 (0.006–0.008)	N.R. (40%)
aq.MeOH fraction	0.03 (0.01–0.04)	0.004 (0.003–0.004)	N.R. (45%)

N.R.: no IC_50_ was observed and maximum cytotoxicity at 5 µg mL^−1^ indicated in brackets.

**Figure 3:**
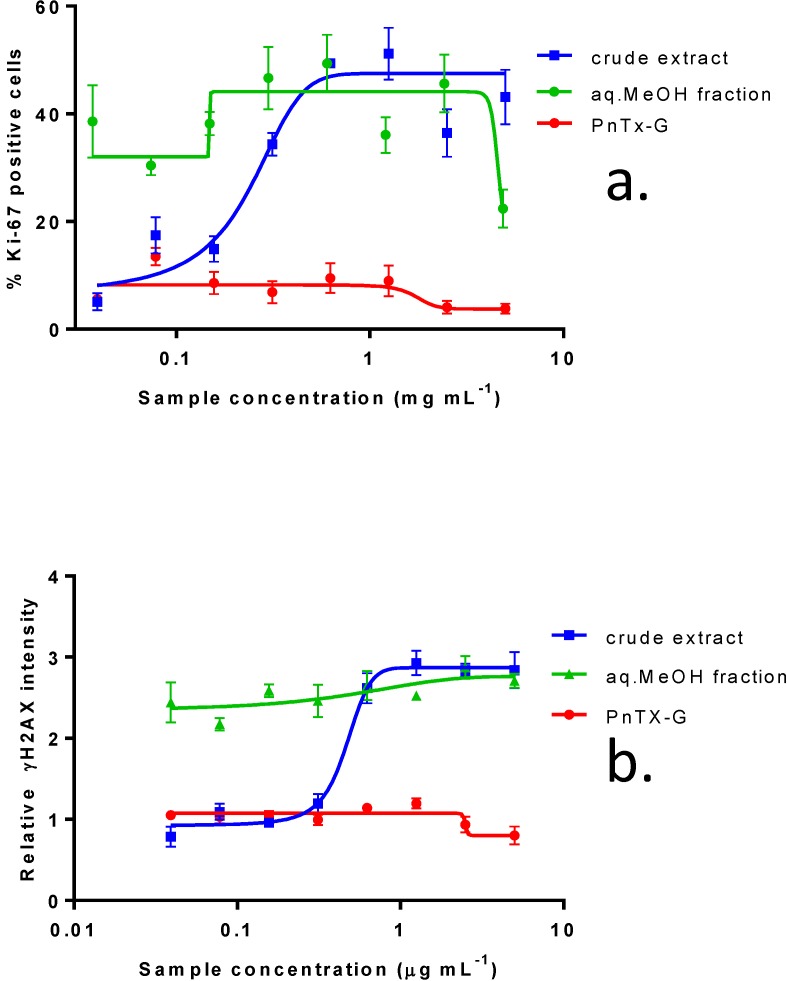
Effect of crude extract of *V. rugosum*, the aqueous methanol fraction and PnTX-G on (**a**) Ki-67 expression and on (**b**) γH2AX phosphorylation in Caco-2 cells. Data are presented as the mean average nuclear intensity of Ki-67 and γH2AX immunofluorescence ± SEM.

Ki-67 was measured as a biomarker of cell proliferation, typically present at specific phases of the cell cycle. Although the crude extract of *V. rugosum* had little effect on Caco-2 viability, there was a significant, dose-dependent increase in Ki-67 positive cells. This effect was even more pronounced for the partially purified aqueous MeOH fraction, where a maximum effect was already observed at the lowest dose level. The distinct punctate pattern of Ki-67 immunofluorescence in cells treated with the crude extract is indicative of a cell cycle arrest in the G1/S phase. This cell cycle arrest was accompanied by a dramatic increase in γH2AX immunofluorescence at mid-range concentrations of crude *V. rugosum* extract and even at the lowest range of the partially purified extract, *i.e.*, in the aqueous MeOH-fraction (Figure 3a,b).

### 2.3. *In Vitro* Effects of Various Fractions of *V. rugosum* Extract

The cytotoxic activities were monitored along the fractionation process on the three different cell lines. 

Five fractions were obtained after fractionation of the aqueous MeOH fraction of the *V. rugosum* extract. Among these fractions, fraction 3 was the only one containing a significant amount of PnTX-G (Table 1). A similar classification of the potency of these fractions according to their IC_50_ was obtained from Neuro2A and KB cells results, as follows: F2 > F3 > F4 > F5 > F1 (Table 3). The most potent fraction—fraction 2—exhibited an IC_50_ of 11 (5–17) and 0.21 (0.18–0.24) ng mL^−1^ on Neuro2A and KB cells, respectively. However, this fraction did not contain PnTX-G. The only fraction containing PnTX-G, fraction 3, showed IC_50_ of 25 and 2.59 ng mL^−1^ on Neuro2A and KB cells, respectively. On undifferentiated Caco-2 cells, no IC_50_ could be determined for any fraction: cytotoxicity increased by ca. 40% except for fraction 4 (25% cytotoxicity), Table 3.

### 2.4. *In Vitro* Effects of Pinnatoxin G

**Figure 4:**
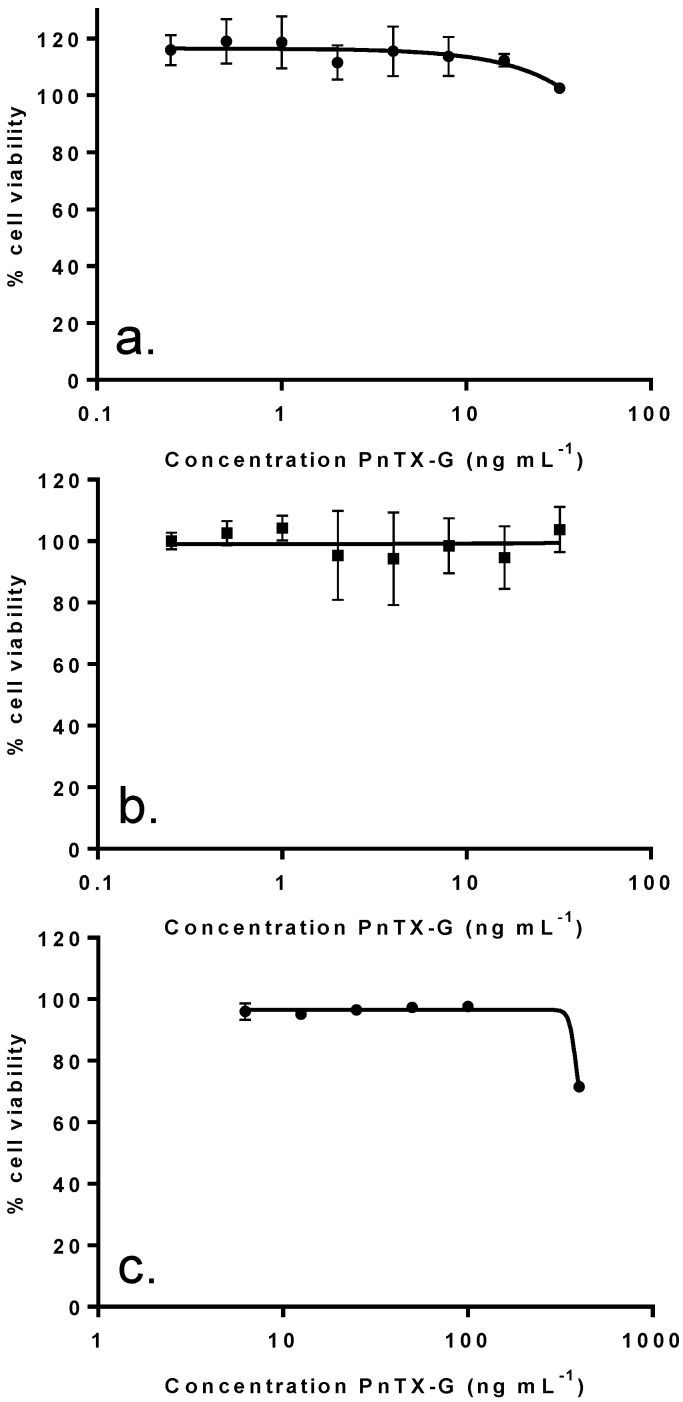
Effect of PnTX-G on Neuro2a cells (**a**), undifferentiated Caco-2 cells (**b**), and KB cells (**c**). The effect was evaluated at a single time exposure: 24 h for Neuro2A, 48 h for undifferentiated Caco-2 and 72 h for KB cells. Data presented (mean ± SEM, in graph **c**, some error bars are smaller than the symbol of the data point) were obtained from two independent experiments (duplicate wells per experiment for Neuro2A and Caco-2, triplicate wells per experiment for KB cells).

In order to determine whether PnTX-G could contribute to the cytotoxicity observed with the crude extract, the pure toxin was tested on the different cell lines. Surprisingly, no cytotoxicity was induced in either Neuro2A cells (24 h exposure) or in undifferentiated Caco-2 cells (48 h exposure) following exposure to PnTX-G up to 32 ng mL^−1^ (Figure 4a,b). Similarly, cell viability of differentiated Caco-2 was not affected by PnTX-G up to 32 ng mL^−1^ (data not shown). In KB cells, higher concentrations up to 400 ng mL^−1^ were tested, however, only slight reductions in KB cell viability (27%) were observed following 72 h of treatment (Figure 4c). In addition, PnTX-G had no effect on either cell cycle progression or DNA damage in undifferentiated Caco-2 cells (Figure 3a,b). 

**Table 3:** Cytotoxicity IC_50_ values (ng mL^−1^) for the 5 fractions obtained using SPE cartridge (*N* = 2).

IC_50_ (ng mL^−1^): median (values obtained with two independent experiments)					
	Fraction Weight (%)	Distribution of PnTX-G (%)	KB cells (72 h of incubation)	Neuro2A cells (24 h of incubation)	Undifferentiated Caco-2 cells (48 h of incubation)
**F1**	4.9	0	1023 (958, 1088)	N.R. (45%)	N.R. (44.5%)
**F2**	50.3	0	0.21 (0.18, 0.24)	11 (5–17)	N.R. (41.6%)
**F3**	26.5	100	2.59 (2.55, 2.63)	25 (44–6)	N.R. (42.6%)
**F4**	4.6	0	53.6 (45.4, 61.8)	575 (760–390)	N.R. (25.2%)
**F5**	13.8	0	62.7 (55.9, 69.5)	1140 (800–1480)	N.R. (41.1%)

N.R.: no IC_50_ was observed and maximum cytotoxicity at 5 µg mL^−1^ indicated in brackets.

**Figure 5:**
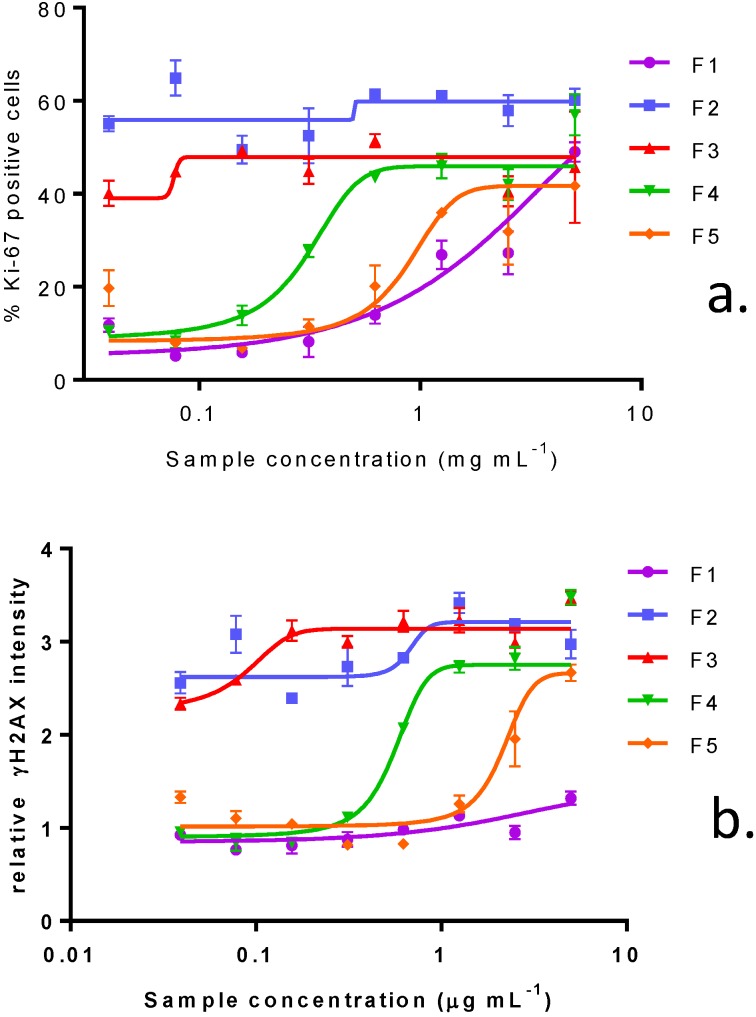
Effect of purified fractions of *V. rugosum* on Ki-67 expression (**a**) and on γH2AX phosphorylation (**b**) in Caco-2 cells. Data are presented as the mean average nuclear intensity of Ki-67 and γH2AX immunofluorescence ± SEM.

**Figure 6:**
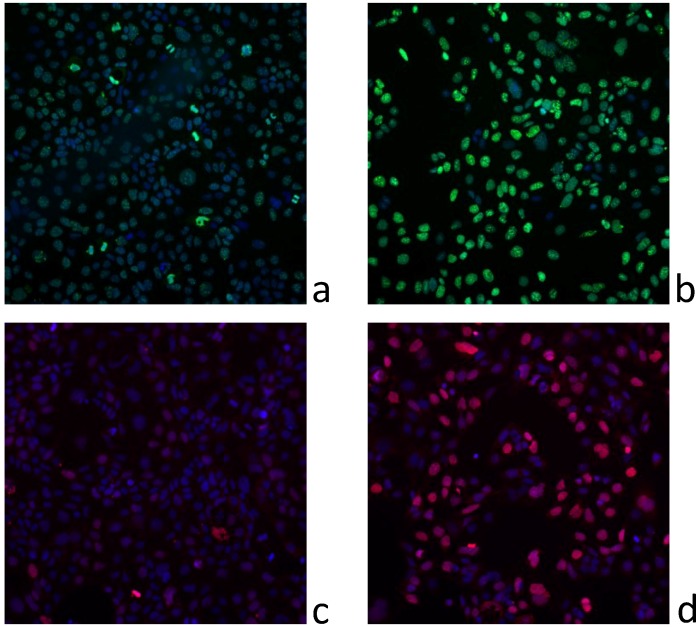
Representative images of (**a**) γH2AX immunofluorescence in untreated Caco-2 cells; (**b**) Caco-2 cells treated with F3 at 156 ng mL^−1^; (**c**) Ki-67 immunofluorescence in untreated Caco-2 cells; (**d**) Caco-2 cells treated with F3 at 156 ng mL^−1^.

In undifferentiated Caco-2 cells, the percentage of Ki-67 positive cells rose dramatically following treatment with purified fractions. This effect was primarily due to an increase in cells demonstrating a distinct punctate nuclear immunostaining, indicative of G1/S cell cycle arrest. Even though the fractions did not induce a large cytotoxicity in Caco-2 cells, in agreement with the cytotoxicity observed in Neuro2A and KB cells, fraction 2 had the greatest effect in promoting cell cycle arrest, followed by F3, F4, F5 and F1 (Figure 5a, Figure 6a,b). The increase in γH2AX immunofluorescence was closely associated with the cell cycle arrest, although appearing at higher concentrations of the purified fractions (Figure 5b, Figure 6c,d).

### 2.5. Dereplication Study

**Figure 7:**
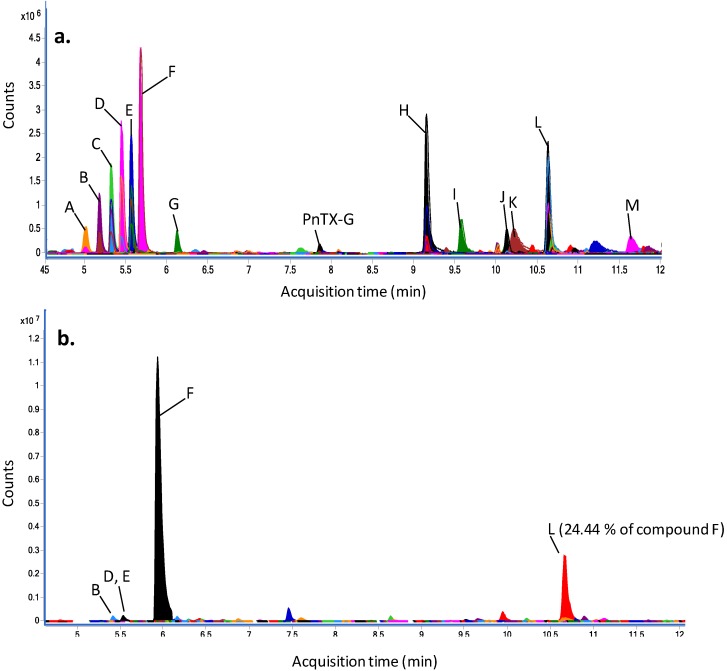
Compounds identified (using the *Molecular-Feature-Extraction* algorithm of the Mass Hunter software) (**a**) in a crude extract of an axenic culture of *V. rugosum*; (**b**) the F2 fraction obtained from a non-axenic culture of *V. rugosum*. The peak identification is detailed in Table 4.

The chemical profile of the crude extract obtained from an axenic culture of *V. rugosum* was investigated using high resolution mass spectrometry in full scan mode. Analyses were carried out in triplicate, and the peaks obtained in the three experiments were dereplicated. PnTX-G could be confirmed on the basis of the retention time, fragmentation and the *m/z* using an in-house database of our laboratory. For dereplication of unknowns, Agilent Mass Hunter software was used to calculate scores which are based both on the closeness of the measured *m/z*-value to the theoretical value (∆ ppm) in a database and on the isotope distribution. Thus, compounds with identical molecular formulas (isobaric compounds) have the same scores. Using the MarinLit™ [21] database, which does not contain retention time information, the most abundant compound (peak F) gave three hits for isobaric compounds: nakijiquinone A, *N*-carboxy-methyl-smenospongine or stachybotrin A, with a very good score of 99.4 (Figure 7a and Table 4). In Table 4, the abundance of each peak was relative to the abundance of the major peak, compound F. Five other compounds could be putatively annotated; however, several major compounds remained unidentified.

**Table 4:** Putative annotation of peaks observed on a chromatogram of an axenic *V. rugosum* crude extract, using the MarinLit™ database (SD = standard deviation).

Peak	Experimental *m/z* [M + H]^+^		Relative abundance of compound F (%)		Putative annotation using MarinLit database	Score (DB)		Diff (DB, ppm)	
	Mean ( *N* = 3)	SD	Mean ( *N* = 3)	SD		Mean ( *N* = 3)	SD	Mean ( *N* = 3)	SD
**A**	393.2102	0.0001	16.74	0.72	unknown	-	-	-	-
**B**	437.2362	0.0002	26.65	0.62	unknown	-	-	-	-
	432.2810	0.0002	9.97	0.47	unknown	-	-	-	-
**C**	481.2622	0.0001	38.61	1.04	unknown	-	-	-	-
	476.3067	0.0001	18.21	1.49	unknown	-	-	-	-
**D**	520.3331	0.0002	33.91	0.50	unknown	-	-	-	-
	525.2886	0.0002	59.92	0.90	unknown	-	-	-	-
**E**	564.3593	0.0001	36.13	3.03	unknown	-	-	-	-
	569.3147	0.0001	59.10	5.14	unknown	-	-	-	-
**F**	613.3408	0.0003	13.70	5.11	unknown	-	-	-	-
	402.2281	0.0003	100.00	100.00	Nakijiquinone A	98.14	1.34	−1.38	0.56
					*N*-carboxy-methyl-smenospongine				
					Stachybotrin				
**G**	370.2748	0.0000	10.83	0.23	*N*-methyl-xestamine D	99.44	0.03	−0.84	0.02
**PnTX-G**	694.4664	0.0003	1.06	0.06	-	-	-	-	-
**H**	343.2958	0.0001	66.03	1.95	unknown	-	-	-	-
	240.2327	0.0001	8.74	0.50	2-hydroxypentadecanoic acid	98.13	0.44	−1.60	0.34
**I**	272.2589	0.0001	20.50	0.70	2-amino-1,3,4-hexadecanetriol; (2 *S*,3*R*,4*R*)-form	99.11	0.21	−1.18	0.14
**J**	692.3843	0.0003	4.04	0.38	unknown	-	-	-	-
**K**	286.1444	0.0002	11.69	0.80	Solanapyrone B; 7ct-Hydroxy, 4′-demethoxy, 4′-amino, 1-aldehyde	99.03	0.71	−1.12	0.53
**L**	453.1680	0.0001	61.23	0.14	7,11-dihydroxy-16-oxo-12-spongien-17-al; (713,1113)-form, Di-Ac	97.96	0.94	−1.44	0.36
					Furcellataepoxylactone				
					Branacenal				
	288.2538	0.0001	9.02	0.52	unknown	-	-	-	-
**M**	402.3575	0.0001	6.97	0.28	unknown	-	-	-	-

Fraction 2 was analysed in the same way, and compounds observed in the crude extract were examined in this fraction (Figure 7b). As in the crude extract, the compound F was the most abundant. Peaks B, D and E were observed in fraction 2 but only in very low abundances: 0.05%, 0.06% and 0.07% of compound F, respectively. The second most abundant compound (peak L, 24.4% of compound F), was putatively annotated as 7,11-dihydroxy-16-oxo-12-spongien-17-al, furcellataepoxylactone or branacenal.

## 3. Discussion

Pinnatoxins are classified as “fast-acting toxins” and studies have demonstrated their acute toxicity to mice by intraperitoneal *i.p.* injection [2,3,4]. More specifically, the LD_50_ of PnTX-G by *i.p.* injection in mice was around 50 µg kg^−1^, with lethality always appearing within less than 30 min [5,16]. PnTX-G also exhibited a high toxicity by the oral route, with an LD_50_ of 150 µg kg^−1^ by gavage, with lethalities appearing within 25–40 min [16]. Similar results were found with a crude extract of a peridinoid dinoflagellate producer of PnTXs: the toxicity to mice was high both by *i.p.* injection (1.33 mg kg^−1^) and by gavage (2.33 mg kg^−1^) [7]. The similarity of the LD_50_s determined for these two routes of administration is quite unusual among cyclic imines. Indeed, it has been reported that gymnodimine was eight times less toxic by gavage than by *i.p.* injection, and desmethyl spirolide C was 18 times less toxic [22]. Therefore, contrary to the other cyclic imines, PnTX-G is likely to have a high oral bioavailability and to be easily absorbed through the GI tract.

In the present study, no inhibition of cell viability was observed with PnTX-G on the Neuro2A and Caco-2 cell lines exposed to concentrations as high as 32 ng mL^−1^ as well as on KB cells exposed to up to 400 ng mL^−1^, even after 72 h of exposure. PnTX-A, -E and -F have been shown to block neuromuscular transmission through the inhibition of nicotinic acetylcholine receptors subtypes [19,20]. These receptors also are the target of PnTX-G [11]. This mechanism of action seemed to be strongly related to the spiro-imine subunit [19]. In our study, no inhibition of cell viability was observed with PnTX-G on the different cell lines, which may suggest the absence of PnTX-sensitive nicotinic acetylcholine receptors in these *in vitro* models.

The comparison of the axenic and non-axenic profiles led to the identification of PnTX-G in both cultures. The fact that *V. rugosum* produces PnTX-G also in axenic conditions, and at levels similar to non-axenic cultures, corroborates that PnTX-G is produced *de novo* by the alga and not by bacterial symbionts or fungal contamination present. Several other compounds were also identified in both axenic and non-axenic cultures, including the compound with a *m/z* of 402.2281 ([M + H]^+^). PnTX-G was exclusively concentrated in fraction 3, which was the second-most potent in terms of cytotoxicity (Table 3). As PnTX-G exhibited no activity on the different models, it could be concluded that other compounds were present and responsible for the toxicity. The compound with the *m/z*-value 402.2281 ([M + H]^+^) as well as some unidentified compounds previously discussed could also be detected in this fraction and may be responsible for all or part of the cytotoxicity exhibited by fraction 3 (see also below). 

Fraction 2 was shown to be the most potent fraction to both Neuro2A and KB cells. This fraction was also the most potent in arresting the cell cycle of undifferentiated Caco-2 cells in the G1/S phase. Occurring at lower concentrations, the cell cycle arrest appears to be the primary mechanism of toxicity. The increase in γH2AX immunofluorescence appears at higher concentrations, and may either indicate genotoxicity, or an increase in the number of apoptotic cells. The chemical profile of fraction 2 suggested the presence of compounds with a mass-to-charge ratio of *m/z* 402.2281 ([M + H]^+^) and with a molecular formula putatively corresponding to three compounds in the MarinLit™ database: nakijiquinone A, stachybotrin A or *N*-carboxy-methyl-smenospongine. Nakijiquinone A and *N*-carboxy-methyl-smenospongine both are sesquiterpenoid amino-quinones [23,24], whereas stachybotrin is an aromatic alkaloid [25]. Interestingly, those three classes of compounds are known to exhibit biological activities, especially cytotoxicity, albeit at rather high concentration. Even if the cytotoxicity is not particularly high (µg mL^−1^-range), the concentration of this compound appears rather high, since a 100-fold higher area was measured for peak F (Table 4) compared to PnTX-G (in the absence of calibration, standards peak areas are indicative of concentration of a compound). Nakijiquinones, isolated from Okinawan marine sponges, exhibited various activities as cytotoxicity against L-1210 murine leukemia and KB cells or weak inhibition of the Her-2/Neu tyrosine kinase receptor [26,27]. Smenospongine, the main representative of this compound group, had also been isolated from marine sponges and induced the differentiation of the leukemia cell line K562 into normal cells [28] and the inhibition of the growth of 39 human solid tumors [29]. Smenospongine and derivatives also induced a G1 arrest in the K562 cells, and apoptosis of other leukemia cell lines HL60 and U937 [30]. Stachybotrin A had initially been isolated from the marine fungus *Stachybotris* sp. Antifungal and antimicrobial activities were identified, as well as cytotoxicity on the epithelial adenocarcinoma A-549 cell line, the breast adenocarcinoma MCF-7 cell line and the colon adenocarcinoma HT-29 cell line [25]. Thus, any of these three compounds could potentially be responsible for the cytotoxicity of the fraction F2 of the *V. rugosum* extract. However, the MarinLit™ database only constitutes a collection of marine natural products while many new marine natural products are being discovered each year [31]. Further in-depth literature screening and eventually full purification of the compound will be necessary for full structure elucidation.

## 4. Experimental Section

### 4.1. Reagents

Methanol (MeOH), acetone, acetonitrile and dichloromethane (DCM) were obtained as HPLC grade solvents from JT Baker and Sigma Aldrich. Hexane, formic acid (puriss quality) and ammonium formate (purity for MS) were obtained from Sigma-Aldrich. Milli-Q water for HPLC was produced in-house using a Milli-Q integral 3 system (Millipore). Pinnatoxin G (PnTX-G) was obtained from the National Research Council of Canada.

### 4.2. *Vulcanodinium rugosum* Growth Conditions

*Vulcanodinium rugosum* (IFR-VRU-01I) was grown during 83 days in flat-bottomed glass flask (10 L) of L1-medium [32]. The strain was maintained at 18 °C, with a photon flux density at 200 µmol m^−2^ s^−1^ and a photoperiod of 16 h of light and 8 h of dark. Sea water (38 psu) was collected from the Mediterranean Sea, filtered and used for making up culture media. Cells were collected by centrifugation (3500*g*, 20 min, 4 °C). 

Axenic cultures were obtained by using antibiotic-antimycotic treatment for 72 h using 10 ml L^−1^ penicillin-streptomycin-amphotericin solution (Gibco) containing 10,000 units penicillin and 10,000 µg mL^−1^ streptomycin sulfate and 25 µg mL^−1^ amphotericin B as Fungizone^®^ in 0.85% saline, followed by transfer to fresh medium.

### 4.3. Extraction, Purification and Fractionation of a Crude Extract of *V. rugosum*

**Figure 8:**
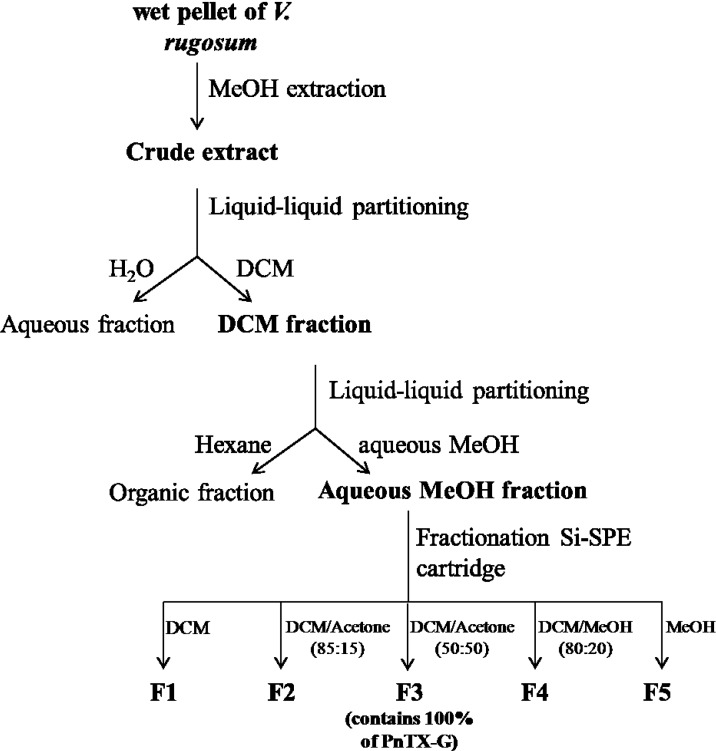
Schematic of the *V. rugosum* pellet purification procedure.

Three successive extractions of wet algal pellet (6.3 g) were carried out using 100% MeOH (20 mL), followed by centrifugation (2500*g*, 10 min, 4 °C). The supernatants were pooled and dried by rotary evaporation, leading to the crude extract (Figure 8). A first purification step was carried out on the crude extract by liquid-liquid partitioning using DCM: distilled water (v/v, 2:1). The DCM phase (300 mL) was rinsed twice with aqueous NaCl 0.1 M (150 mL) and centrifugation (2500× *g*, 10 min, 4 °C). The supernatants were pooled and dried by rotary evaporation, leading to the DCM fraction. A second purification step was carried out on the DCM fraction by liquid-liquid partitioning using aqueous MeOH 70% (aqueous MeOH) and hexane in proportions 2/1. The aqueous MeOH phase (150 mL) was rinsed twice with hexane (50 mL), followed by centrifugation (2500g, 10 min, 4 °C). The supernatants were pooled and dried by rotary evaporation, leading to the aqueous MeOH fraction. A solid-phase extraction (SPE) was then carried out for fractionation of the aqueous MeOH fraction. SPE cartridges were silica-based (SPE UPTI-CLEAN SI-S 500/3, Interchrom). The extractions were carried out using five elution solvents: DCM 100%, DCM/Acetone (85:15), DCM/Acetone (50:50), DCM/MeOH (80:20) and MeOH 100%.

For analysis of an axenic culture of *V. rugosum*, 200 mL of batch culture were sacrificed 2 weeks after axenisation of the main culture. Cells were collected using both glass beads and a scraper, with subsequent centrifugation of the wet biomass. The cell pellet (276 mg) was extracted in triplicate with MeOH (5 mL each) and taken up in 1 mL MeOH prior to further dilutions. Dilutions of this solution were analysed by triple-stage quadrupole analysis for quantitation of PnTX-G and by quadrupole/time-of-flight mass spectrometry for metabolomic assessment of the algal contents.

### 4.4. Quantification of PnTX-G Using a Triple Stage Quadrupole Mass Spectrometer

Analysis of pinnatoxins was performed on a UFLC (model Nexera, Shimadzu) coupled to a triple-quadrupole mass spectrometer (5500Qtrap, AB Sciex). Chromatography was performed on a Hyperclone MOS C8 column (50 × 2.0 mm, 3 µm), with a C8 guard column (4 × 2.0 mm, 3 µm, Phenomenex). A binary mobile phase was used, phase A (100% aqueous) and phase B (95% aqueous acetonitrile), both containing 2 mM ammonium formate and 50 mM formic acid. The flow rate was 0.2 mL min^−1^ and injection volume was 5 µL. The column and sample temperatures were 25 °C and 4 °C, respectively. A gradient elution was employed, starting with 30% B, rising to 95% B over 2.5 min, held for 5 min, then decreased to 30% B in 0.1 min and held for 2.5 min to equilibrate the system.

The LC-MS/MS system was used in positive ionization and multiple reaction monitoring (MRM) mode, with the following three transitions per compound: PnTX-G *m/z* 694.4→676.4, 694.4→458.3 and 694.4→164.1; PnTX-A *m/z* 712.4→694.4, 712.4→458.3 and 712.4→164.1, PnTX-B and C, *m/z* 741.4→723.5, 741.4→458.3 and 741.4→164.1; PnTX-D, *m/z* 782.4→764.4 782.4→488.3 and 782.4→164.1; PnTX-E, *m/z* 784.4→764.4 784.4→488.3 and 784.4→164.1; PnTX F, *m/z* 766.4→738.4, 766.4→488.3 and 766.4→164.1; pteriatoxins A, B and C *m/z* 831.5→787.5, 831.5→458.3 and 831.5→164.1. The most intense transition, giving the product ion *m/z* 164.1, was used to quantify toxins. All toxins were quantified against the PnTX-G standard from NRC, assuming that all analogues had the same response factor as PnTX-G.

The ESI interface was operated using the following parameters: curtain gas 30 psi, temperature: 650 °C, gas1 50 psi; gas2 60 psi, ion spray voltage 5500 V. For detection, parameters were as follows: the declustering potential was 30 V and the entrance potential 10 V. Three collision energies were applied (53, 59 and 67 eV) with collision cell exit potentials of 10, 6 and 10 V for transitions 1, 2 and 3 of each compound, respectively. 

The limit of detection (LOD) was determined from the signal-to-noise ratio of the lowest standard injected after injection of 20 samples, using automated calculation in the Analyst 1.5.1 software of Applied Biosystems: This algorithm calculates the LOD as the sample giving a signal height 3 times the standard deviation of the noise. The LOD (5 µL injected) was 250 fg on the column.

### 4.5. Growth Conditions of the Different Cell Lines

Different cell lines were chosen for this study: Neuro2A, undifferentiated and differentiated Caco-2 and KB cells. Neuro2A and Caco-2 cell lines derived from the nervous system and intestine, respectively, organs that are known as targets for some phycotoxins. These two cell lines, as well as KB cells, have been previously used to study the detection of algal toxins in shellfish extracts [33,34,35,36]. Furthermore, KB cells are a rugged cell line frequently used in our laboratory.

Neuro2A cells: The mouse Neuro2A neuroblastoma cell line (ATCC CCL-131, passages 11–50) was cultured in RPMI 1640-Glutamax containing 2 g L^−1^ glucose, and supplemented with 10% fetal calf serum (FCS), 1 mM sodium pyruvate, 50 U mL^−1^ penicillin and 50 µg mL^−1^ streptomycin. The cell line was routinely grown in 75 cm^2^ flasks at 37 °C and 5% CO_2_ and detached mechanically for sub-culturing.

Undifferentiated Caco-2 cells: The human Caco-2 colorectal adenocarcinoma cell line (ATCC HTB-37, passages 18–40) were cultured in MEM-Glutamax containing 1 g L^−1^ glucose and supplemented with 10% FCS, 50 U mL^−1^ penicillin and 50 µg mL^−1^ streptomycin and 1% non-essential amino acids. The cell line was routinely grown in 75 cm^2^ flasks at 37 °C and 5% CO_2_ and passaged twice a week by trypsinization prior to cell centrifugation (5 min, 136 g).

Differentiated Caco-2 cells: To obtain differentiated Caco-2 cells, 60,000 cells cm^2^ were seeded in 48 wells plates with 200 µL per well of medium as described previously. Medium was renewed each 2–3 days. Caco-2 cells reached confluence after 3 days and were differentiated 21 days after.

KB cells: The KB cells were maintained at 37 °C in a 95% air, 5% CO_2_ atmosphere in Eagle’s basal medium (Sigma-Aldrich) supplemented with 5% (v/v) of FCS and a mix leading to final concentrations of 400 nM l-glutamine, 20 U mL^−1^ penicillin-G and 100 µg mL^−1^ streptomycin (Sigma-Aldrich). Cells were grown in culture flasks (Dutscher) until cell confluence. 

### 4.6. Cytotoxicity Assays

Neuro2A and Caco-2 cells: Except for differentiated Caco-2, cells were seeded in 96-well plates at a density of 50,000 cells 100 µL^−1^ well^−1^ (for undifferentiated Caco-2) and 20,000 cells 100 µL^−1^ well^−1^ (for Neuro2A) 24 h prior to treatment. After removing the medium, cells were exposed in duplicate to the compound in serum free-medium (100 µL well^−1^) for 24 or 48 h, as indicated. The template of each 96 well plate was designed by omitting marginal rows and columns; six wells were used for the control and six wells for the vehicle control. The vehicle control (VeC) contained 5% methanol, the highest concentration of methanol used and which was previously shown to induce no cytotoxicity [34]. Differentiated Caco-2 cells were treated in 48 wells plates with serum free-medium (200 µL well^−1^) for 48 h.

KB cells: Cells were rinsed with Dulbecco’s phosphate buffered saline (Sigma-Aldrich), removed from culture flasks by treatment with trypsin (Sigma-Aldrich), enumerated and diluted with fresh medium. Inoculation of microplates was different for observations at 24, 48 and 72 h or to the single observation at 72 h. For the study with different time points, cells were seeded at a density of 70,000 cells 100 µL^−1^ well^−1^ and plates were incubated for 72 h. Medium culture was then removed from each well and replaced by 50 µL of fresh medium. For the single observation, cells were seeded at a density of 200,000 cells 50 µL^−1^ well^−1^ and plates were incubated 48 h. The template of each 96 well plate was designed by omitting marginal rows and columns. For the two types of experiments, test samples were prepared in MeOH, diluted 10-fold in culture medium and 50 µL of this diluted test solution were added to each well, reaching a final concentration of 5% methanol. Concentrations were tested in triplicate, and experiments were carried out in duplicate. Culture medium with 5% MeOH was used as negative control.

### 4.7. Assessment of the Cell Viability

Cytotoxicity testing for Neuro2A and undifferentiated Caco-2 cells followed the conditions set up in Sérandour *et al.* (2011) [34]. Only a short duration of exposure was selected for Neuro2A while exposure on Caco-2 and KB cells was increased up to 72 h due to the longer cell doubling time of these two models (20 h and 48 h, respectively).

For Neuro2A and Caco-2 cells, the cytotoxicity was measured using the 3-(4,5-dimethylthiazol-2-yl)-2,5-diphenyltetrazolium bromide (MTT) assay. After treatment, the medium was replaced by a FCS-free medium containing 0.5 mg mL^−1^ MTT (Sigma) for 2 h at 37 °C. The medium was discarded prior to the addition of 0.1 N HCl-acidified isopropanol to dissolve the formazan. The absorbance was read at 570 nm and was expressed as the percentage of mean absorbance (*N* = 2) in VeC (100% viability).

For KB cells, after 24, 48 or 72 h of incubation at 37 °C, 50 µL of a mix of solution 1 and 2 of a kit based on the 2,3-bis(2-methoxy-4-nitro-5-sulfophenyl)-5-[(phenylamino)carbonyl]-2*H*-tetrazolium(XTT-based kit, Roche) were added to each well, followed by an incubation of 4 h at 37 °C. Plates were gently shaken and read at 405 nm and 630 nm with a microplate reader (EL800, Bio-Tek). The absorption values for each extract were averaged and the averages were then expressed as a percentage, relative to the solvent control.

### 4.8. IC_50_ Determination

Inhibition curves for Neuro2A and Caco-2 cells were modeled using a nonlinear regression analysis (GraphPad Prism, version 5.0c, San Diego, CA, USA). Concentrations at which cell growth was inhibited to 50% (IC_50_s) and 95% confidence intervals were also calculated with the same model. The model did not fit with the curve of KB cells viability, so IC_50_s were calculated using linear interpolation of the two points surrounding 50% inhibition.

### 4.9. DNA Damage and Cell Cycle Progression Measurements

Following 24 h treatment, undifferentiated Caco-2 cells in 96-well plates were fixed with 4% formaldehyde and permeabilised with 0.2% Triton X-100. After 3 washes with PBS-tween 20 (0.05%), cells were incubated overnight with an anti-γH2AX mouse monoclonal antibody (Thermo MA1-2022; 1:1500), or a rabbit anti Ki-67 (Abcam ab15,580; 1:1000). Histone H2AX is phosphorylated into γH2AX after DNA damage and Ki-67 is a marker of cell proliferation. Cells were then washed 3 times with PBS-tween and incubated with Dylight 550-conjugated goat anti-mouse or Dylight 488-conjugated goat anti-rabbit secondary antibodies (1:1000) for 45 min. Nuclei were stained with DAPI. The mean average fluorescence intensity was quantified using an Arrayscan VTi high content screening microscope using the Target Activation algorithm. The average nuclear intensity was calculated from ten fields per well, three wells per condition. Two independent experimentations were carried out.

### 4.10. Dereplication Study Using a Quadrupole-Time-of-Flight Hybrid Mass Spectrometer

As the cytotoxicity study suggested the presence of cytotoxic compounds different from PnTX-G, an in-depth study was undertaken using high resolution mass spectrometry (HRMS). 

Aliquots (5 µL) of each sample were separated on a Kinetex 1.7 µm C18 100Å (Phenomenex) column (150 × 2.1 mm) maintained at 40 °C, using an Agilent 1290 Infinity LC system with a gradient mobile phase (0.5 mL min^−1^) comprising 0.1% aqueous acetic acid (A) and acetonitrile containing 0.1% acetic acid (B). The gradient was as follows: 5% B from 0 to 2.4 min, raise to 25% B from 2.4 to 4.5 min, then raise to 30% B from 4.5 to 11 min, finally raise to 100% B from 11 to 14 min and maintain until 16.5 min, subsequently decrease to 5% B until 20 min and maintain at 5% B until 25 min. The eluent was directly introduced into the mass spectrometer by electrospray. Mass spectrometry was performed on a 6540 UHD Q-TOF mass spectrometer (Agilent Technologies, Waldbronn, Germany), operating in positive ion mode. The capillary voltage, fragmentor voltage and skimmer were set to 3900, 150 and 60 V, respectively. The sheath gas was at 350 °C (12 mL min^−1^) and the drying gas at 175 °C (5 mL min^−1^) and nebulizer 43 psi. Nitrogen was used as collision gas. Mass spectra were acquired in full scan analysis over an *m/z* range of 50–1700 using extended dynamic range and storage in centroid mode. To maintain mass accuracy throughout the run, a reference mass solution containing reference ions 121.0508 and 922.0097 was used in the positive ionization mode. The correction of mass axis using these reference ions typically results in a mass accuracy of 1 ppm in full scan mode and 2 ppm in fragmentation mode. Data station operating software was MassHunter Workstation Software (versionB.05).

Metabolomic fingerprints were deconvoluted to allow the conversion of the three-dimensional raw data (*m/z*, retention time, ion current) to time- and mass-aligned chromatographic peaks with associated peak areas. Data were processed using MassHunter Qualitative Analysis software (Agilent Technologies) where compounds were extracted from the raw data using the Molecular Feature Extractor (MFE) algorithm.

*Molecular feature extractor.* The MFE algorithm is a compound finding technique that locates individual sample components (molecular features), even when chromatograms are complex and compounds are not well resolved chromatographically. MFE locates ions that are covariant (rise and fall together in abundance) and the algorithm uses the accuracy of the mass measurements to group related ions—related by charge-state envelope, isotopic distribution, and/or the presence of adducts and dimers. It assigns multiple species (ions) that are related to the same neutral molecule (for example, ions representing multiple charge states or adducts of the same neutral molecule) to a single compound that is referred to as a feature. Using this approach, the MFE algorithm can locate multiple compounds at any given retention time, *i.e.*, also in a single peak [37].

The result is a compound table with associated chromatograms and pure spectra, ready for further analysis. Subsequently, samples were processed using MassProfiler software (Agilent Technologies) and compound identification was performed using an in-house database and the MarinLit^TM^ database.

To ensure reproducibility and precision, analyses were carried out in triplicate.

## 5. Conclusions

PnTX-G is a marine toxin inducing strong neurological symptoms in mice but this study revealed that it is not a potent cytotoxic compound, using the Neuro2A, Caco-2and KB cell lines. However, a crude extract of a culture of *V. rugosum* induced a reduction of the cell viability of those three different cell lines, as well as cell cycle arrest accompanied by DNA damage. A crude extract was fractionated and the fraction exhibiting the more potent *in vitro* activity did not contain PnTX-G. The dereplication of the crude extract and fraction revealed that these activities could be induced by compounds such as nakijiquinone a, *N*-carboxy-methyl-smenospongine or stachybotrin A, which have previously been reported to be cytotoxic. Further investigations will be necessary to identify and purify compounds responsible for the activity of *V. rugosum* crude extracts and fractions. 
